# The Obscure World of Integrative and Mobilizable Elements, Highly Widespread Elements that Pirate Bacterial Conjugative Systems

**DOI:** 10.3390/genes8110337

**Published:** 2017-11-22

**Authors:** Gérard Guédon, Virginie Libante, Charles Coluzzi, Sophie Payot, Nathalie Leblond-Bourget

**Affiliations:** DynAMic, Université de Lorraine, INRA, 54506 Vandœuvre-lès-Nancy, France; virginie.libante@univ-lorraine.fr (V.L.); charles.coluzzi@univ-lorraine.fr (C.C.); sophie.payot-lacroix@inra.fr (S.P.)

**Keywords:** mobilization, gene transfer, conjugation, integrative mobilizable element, mobilizable transposon, mobile genomic island, antibiotic resistance

## Abstract

Conjugation is a key mechanism of bacterial evolution that involves mobile genetic elements. Recent findings indicated that the main actors of conjugative transfer are not the well-known conjugative or mobilizable plasmids but are the integrated elements. This paper reviews current knowledge on “integrative and mobilizable elements” (IMEs) that have recently been shown to be highly diverse and highly widespread but are still rarely described. IMEs encode their own excision and integration and use the conjugation machinery of unrelated co-resident conjugative element for their own transfer. Recent studies revealed a much more complex and much more diverse lifecycle than initially thought. Besides their main transmission as integrated elements, IMEs probably use plasmid-like strategies to ensure their maintenance after excision. Their interaction with conjugative elements reveals not only harmless hitchhikers but also hunters that use conjugative elements as target for their integration or harmful parasites that subvert the conjugative apparatus of incoming elements to invade cells that harbor them. IMEs carry genes conferring various functions, such as resistance to antibiotics, that can enhance the fitness of their hosts and that contribute to their maintenance in bacterial populations. Taken as a whole, IMEs are probably major contributors to bacterial evolution.

## 1. Introduction

Conjugation is a key mechanism of horizontal gene transfer between bacteria and therefore a major contributor of plasticity and evolution of prokaryotic genomes [1,2]. Conjugation allows the transfer of DNA between bacterial cells in close contact [3] and is mediated by different types of mobile genetic elements (MGEs) [4]. Among them, the conjugative plasmids have been discovered a long time ago and are extensively studied since (i) they are easily identified; (ii) they are widely distributed in bacteria and (iii) they massively contribute to the dissemination of pathogenesis and antibiotic resistance determinants. As plasmids, these elements are extra-chromosomal double stranded DNA (dsDNA) molecules that control their own replication ensuring their vertical transmission and, as conjugative elements, they encode all functions needed for their horizontal transfer [5]. Two mechanisms of transfer are used by conjugative plasmids. The less complex mechanism involves only one protein (TraB) that mediates dsDNA transfer and is related to FtsK, a protein involved in dsDNA translocation during cell division. [6]. This mechanism is restricted to multicellular Actinobacteria such as *Streptomyces* spp. and their relatives. The second transfer system promotes single-stranded DNA (ssDNA) transfer and concerns many plasmids from diverse bacterial phyla (e.g., Proteobacteria, Firmicutes, Bacteroidetes, Actinobacteria) [5].

Over the years, the understanding of the mechanism of ssDNA transfer of plasmids from Proteobacteria has made huge advances, while those of other bacterial divisions remain poorly known. The conjugative apparatus includes a relaxase, a mating pair formation system (MPF) and a coupling protein (CP). The conjugation transfer can be divided in few steps. (i) The attachment of the donor cell to the recipient is mediated mainly by cell surface pili and/or adhesins. (ii) The processing of the DNA is ensured by the relaxase, possibly with accessory components encoded by the element. The relaxase recognizes, cleaves and covalently attaches to the origin of transfer (*oriT*) on the DNA to be transferred. Six distinct families of “canonical” relaxases (MobC, MobF, MobH, MobP, MobQ and MobV) are encoded by conjugative plasmids from Proteobacteria [7]. (iii) The passage of the ssDNA from the donor cell to the recipient cell is initiated by the interaction between the ssDNA-relaxase complex (relaxasome) and the CP that belongs to the VirD4 family and is thought to be a DNA transporter [3]. The CP and the MPF both constitute a spanning-membrane multi-protein complex named a type IV secretion system (T4SS). The T4SS encoded by the element ensures the translocation of the relaxase-tethered DNA from the donor to the recipient bacteria. The Gram-T4SS is composed of three parts, an inner complex associated to the inner membrane, an outer complex and the pilus [3,8]. Gram + T4SSs do not include an outer complex nor a pilus [9,10]. (iv) Alongside with its transfer, plasmid ssDNA is replicated in both the donor and the recipient cells. The relaxase is involved in the initiation of rolling-circle-like plasmid replication and recircularization of plasmid DNA in the donor and recipient [11]. Overall, the canonical relaxases are involved in the initiation of both the transfer and the replication concomitant to the ssDNA transfer. However, they are not involved in the maintenance of the plasmid as an extra-chromosomal form.

More recently, besides conjugative plasmids, another type of conjugative elements, the integrative and conjugative elements (ICEs), was identified in various bacteria [12,13]. ICEs are integrated into the chromosome (or a plasmid) of their host. They are replicated and inherited as a part of these replicons during cell division. Globally, ICEs, also known as conjugative transposons, can be defined as elements encoding their excision, their transfer by conjugation and their integration, regardless of mechanisms [12]. The integration and the excision of ICEs, like those of integrated prophages, can rely on tyrosine recombinases, serine recombinases or DDE transposases. Although less well known than conjugative plasmids, recent analyses of bacterial genomes reveal a very high prevalence of ICEs and suggest that their abundance largely exceeds that of conjugative plasmids [1,12,14,15].

The mechanism of ICE transport is thought to be similar to that of conjugative plasmids although less documented [16]. Except the ICEs of *Streptomyces* that transfer by a dsDNA mechanism, all other ICEs transfer by a ssDNA mechanism. As for ssDNA transfer of plasmids, the transfer of these ICEs involves a relaxase, a CP and a MPF. However, the study of ICEs from Firmicutes reveal that if many encode canonical relaxases, many others encode non-canonical relaxases (MobT). MobT relaxases are related to initiators of rolling circle replication (RCR) that belong to Rep_Trans family and that are involved in the maintenance of many plasmids from Firmicutes [14,17]. The MobT-encoding ICEs encode a non-canonical CP, related to the one of the plasmid pCW3 from the Firmicute *Clostridium perfringens* [18,19] and more distantly related to FtsK, or the TraB protein involved in dsDNA conjugative systems from Actinobacteria [14,17]. Although the maintenance of ICE is essentially based on their integrated form, recent data suggest that their replication as a circular form has a significant role in their maintenance (for reviews, see [12,20,21]). Like all other bacterial MGEs [22], ICEs have a modular structure, i.e., the genes involved in the same biological function (such as conjugation or integration/excision) are physically linked in a module [12,23]. Multiple exchanges of integration/excision and/or conjugation modules between ICEs were reported [12,14], showing that ICEs mainly evolved by module acquisition, loss or exchanges. 

The impact of conjugation goes, however, far beyond the transfer of self-transmissible elements. For example, ICEs can promote the transfer of DNA sequences physically linked to the element (*cis*-mobilization), leading to the transfer of chromosomal genes through an Hfr-like mechanism, or of non-autonomous MGEs carried by the ICE, carrying the ICE, or integrated in tandem (see [12], for a review). The impact of conjugation also extends to mobilization *in trans*, a process in which a non-self-transmissible element hijacks the conjugative apparatus of a co-resident conjugative element to promote its own transfer (for reviews [12,24]). The mechanism of *trans*-mobilization is well documented for plasmids from Proteobacteria. Mobilizable plasmids carry their own *oriT*, relaxase gene and frequently gene(s) encoding relaxosome accessory factor(s) but lack genes required for T4SS formation. Therefore, these elements are mobile in cells that carry other MGE(s) encoding a compatible T4SS [11].

Although less known than mobilizable plasmids and rarely identified, a class of integrated MGEs, the integrative and mobilizable elements (IMEs), can be mobilized *in trans* by conjugative elements. The IMEs, also known as mobilizable transposons, can be defined as elements that encode their own excision and integration regardless of their mechanism and/or specificity of integration and that are able to hijack or subvert the mating apparatus of related or unrelated conjugative elements, regardless of mechanism [12]. All the very few documented IMEs discovered before 2005 display mobilization mechanisms similar to those of canonical mobilizable plasmids. Indeed, they carry an *oriT*, encode a canonical relaxase and possibly other proteins of the relaxasome but no protein of the T4SS (including CP). Therefore, these canonical IMEs display all the DNA processing functions but entirely rely on the T4SS of the helper conjugative element.

However, starting from 2010, various non-canonical IMEs have been discovered, such as elements devoid of any relaxase, elements encoding some T4SS proteins that reshape the mating apparatus of conjugative plasmids to promote their own transfer, or putative elements that encode a non-canonical CP and/or a non-canonical relaxase [25,26,27]. Globally, recent data reveal that the diversity of IMEs goes far beyond that previously observed and that IMEs use many different mobilization strategies. Furthermore, they also suggest that, although few IMEs are known, these elements are even more widespread than ICEs and therefore that known IMEs are only the tip of the iceberg.

The conjugative and mobilizable elements, including IMEs, are known to be major vehicles for acquisition of a broad spectrum of antibiotic resistance genes among bacteria and many other genes that can be advantageous for their host. This review will address the diversity of IME maintenance, transfer mechanisms and interactions with the conjugative elements. It will also update our knowledge about adaptive functions carried by IMEs and therefore will address the question of the impact of IMEs in the spread of antibiotic resistance and more largely of their role in bacterial adaptation to environment.

## 2. Maintenance of IMEs

### 2.1. Integration and Excision

Like integrated prophages and ICEs, all IMEs encode integrases that catalyse their excision and integration. Most of them also encode factors that probably interact with the integrase. In a large majority of IMEs, the genes involved in integration/excision are clustered in a module that corresponds to one end of the integrated element.

Many IMEs encode a single site-specific integrase belonging to the family of tyrosine recombinases (Table 1).

**Figure 1:**
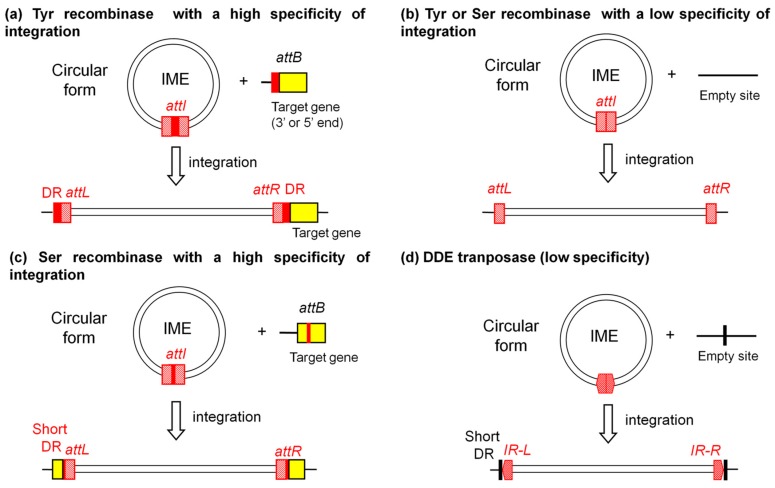
Schematic representation of the integration of IMEs encoding: (**a**) a tyrosine recombinase allowing high specific integration; (**b**) a tyrosine or serine recombinase with a low specificity of integration; (**c**) serine recombinase with a high specificity of integration and (**d**) DDE transposase. The integration is mediated by the recombinase or the DDE transposase. Most IMEs encoding a recombinase integrate in a specific *attB* site that corresponds to the 5′ or 3′ end of a gene (tyrosine recombinase) or is located within a gene (serine recombinase). The recombination between two short identical or almost identical sequences carried by *attI* and *attB* sites leads to DRs flanking the integrated IME. Some IMEs encoding a serine or tyrosine recombinase and the two known IMEs encoding a DDE transposase have a low specificity of integration and therefore do no integrate in a specific *attB* site. The integration of IMEs encoding DDE transposases leads to duplication of the targeted sequence leading to short DRs flanking the integrated IME. Attachment sites are drawn as rectangles: in red with motif, arms of the left (*attL*) and right (*attR*) attachment sites and corresponding arms of *attI*; in red, identical sequences found in the *attB*, *attL*, *attR* and *attI* sites. The left and right inverted repeats (IR-L and IR-R) found in IMEs encoding a DDE transposase are drawn as red arrows. The yellow rectangles indicate target gene and include a copy of the sequence found in all *att* sites (red rectangle).

The tyrosine recombinases recognize the *attR* and *attL* attachment sites flanking the IMEs and catalyse the IME excision by a site-specific recombination that generally takes place between 8–60 bp short direct repeats included in the *att* sites (Figure 1). This leads to an extra-chromosomal circular IME carrying an *attI* site and a chromosome devoid of the IME carrying an *attB* site (Figure 1). After transfer, the recombinase catalyses the integration of the IME by recombination between the 8–60 bp sequences included in both the *attI* site and the *attB* site present in the chromosome of the transconjugant. As the IME is assumed to replicate by rolling circle during the transfer, the IME probably reintegrates in the chromosome of the donor cell after transfer. Recently, the analysis of the integration/excision modules of three putative IMEs, MTn*Pi1*, MTn*Pi2* and MTn*Pi3* from *Prevotella intermedia*, which can excise from the chromosome, indicated that each of them encodes a tandem of two distantly related tyrosine recombinases instead of a single tyrosine integrase [44].

Up to 2010, only very few IMEs from *Clostridia*, i.e., Tn*4451* and its very close relatives [33,54] were found to encode an integrase belonging to the family of serine recombinase, a family of proteins that catalyse recombination by mechanisms unrelated to the one of tyrosine recombinases. More recent genome analyses, or re-analyses of published elements that were not initially recognized as IMEs, allow the identification of various IMEs encoding a single serine recombinase in Firmicutes [12,26,32]. All these IMEs are flanked by very short direct repeats included in their putative *attL* and *attR* sites (Figure 1). The excision and/or integration has been described so far only for very few serine-recombinase encoding IMEs (Table 1). The excision and integration of the best studied of these IMEs, Tn*4451*, depend on a serine integrase that catalyses a recombination event between 2-bp identical sequences harboured by its *att* sites [55].

Up to now, only two IMEs, MTn*Sag1* from *Streptococcus agalactiae* and the related element tIS*Cpe8* from *C. perfringens*, encode an integrase belonging to the family of IS*1595* DDE transposases [28,29]. The boundaries of these IMEs share common typical features with those of all mobile genetic elements encoding DDE transposases (i.e., most insertion sequences and transposons, some integrated prophages and ICEs). Indeed, these IMEs exhibit 25 bp-terminal inverted repeats that are assumed to be recognized by the transposase. Their insertion within the chromosome generates 8 bp-direct repeats that flank the IME and correspond to a duplication of the target DNA (Figure 1). The study of MTn*Sag1* in *Escherichia coli* revealed a circular intermediate [28]. It is likely that the mechanism of excision/integration of MT*nSag1* and tIS*Cpe8* during conjugation is similar to the one suggested for insertion sequences encoding a related DDE transposase, i.e., the insertion sequences belonging to IS*1595* family [56]. Hence, the excision of these elements might be replicative, leaving a copy of the element integrated in the original site and generating a double-strand circular copy that would be involved in conjugation.

For prophages and ICEs encoding a tyrosine integrase, a recombination directionality factor (RDF) encoded by the element, also known as excisionase, generally helps to reverse the direction of the recombination towards excision [57]. Such RDFs have also been identified (or proposed) for most IMEs encoding tyrosine integrases [26,38,41,58,59,60]. Besides a RDF and a tyrosine integrase, an additional protein encoded by the IME Tn*4555* is required for its optimal excision from *Bacteroides fragilis* genome [59]. In the same way, in addition to a RDF and a tyrosine integrase, the excision of the IME NBU1 from *Bacteroides uniformis* requires a large region (including two genes, *oriT* and a part of the relaxase gene) [61]. In contrast and like most serine recombinases, TndX encoded by Tn*4451* from *Clostridioides difficile*, the only well characterized IME encoding a serine-recombinase, efficiently catalyses both integration and excision without RDF involvement [55]. Although no RDF has been identified or proposed for any known IME encoding serine integrases, it cannot be excluded that RDFs would be involved in excision of some IMEs, as observed for various prophages [62].

Most IMEs integrate in specific target sites (Table 1). Indeed, most IMEs encoding a single tyrosine recombinase integrate in a large array of specific sites including the 3′ end, or part of, various tRNA genes, the 3′ end of genes encoding various housekeeping proteins (such as ribosomal proteins) or their 5′ end (more rarely) (Table 1). As one of the direct repeats flanking IMEs includes one end of the target gene, their integration does not disrupt it. Some IMEs, encoding closely related tyrosine integrases, were recently found to specifically integrate in the conserved *nic* site of *oriT* of very distantly related ICEs (Tn*916* and ICE*St3*-related elements) in *S. agalactiae* [48,63]. As observed for tyrosine integrase-encoding IMEs, most IMEs encoding a serine recombinase integrate in specific sites (Table 1 and Figure 1). However, recent works on IMEs encoding site-specific serine recombinases showed that their integration leads to the disruption of genes that are dispensable for the bacterial host (Table 1, [26]). Three IMEs found in three different species of *Streptococcus* and encoding related serine integrases are integrated in the same site within *rumA*. This gene encodes a widespread rRNA methyltransferase. In *E. coli*, the deletion of *rumA* has little effect on growth or on the fidelity of translation but alters the susceptibility of the ribosomes to some antibiotics [64]. Furthermore, various IMEs from Firmicutes integrate in specific sites within conserved genes from ICEs belonging to the Tn*5252* superfamily, e.g. in *traG* that encodes the VirD4 coupling protein [12,26,32]. The high integration specificity of most IMEs does not exclude the integration in other sites. Indeed, the IMEs NBU1 from *B. uniformis* and SGI1 from *Salmonella enterica* integrate at low frequency into alternative or secondary *att* sites, especially if the preferred site is absent [65,66].

Various IMEs have lower integration specificity (Figure 1). For instance, Tn*4555*, an IME from *B. fragilis* encoding a single tyrosine integrase, preferentially integrates upstream or downstream of a 207-bp direct repeat sequence of a 589-bp genomic locus of *B. fragilis* [67]. After the deletion of *tnpA*, the gene located upstream of the integrase gene, the insertion pattern is essentially random, suggesting that the integrase itself has a very low specificity [68]. Other IMEs, with low integration specificity, prefer AT-rich regions. These elements include Tn*5520* from *B. fragilis* (an IME encoding a single tyrosine integrase) [50], the three IMEs MTn*Pi1*, MTn*Pi2* and MTn*Pi3* from *P. intermedia* encoding a duo of tyrosine integrases [44] and the two IMEs MTn*Sag1* from *S. agalactiae* [28] and tIS*Cpe8* from *C. perfringens* encoding a DDE transposase [29]. The last example is the IME Tn*4451* from *C. perfringens* and its relatives whose serine integrases also lead to a low specificity of integration of these elements [33].

Most IMEs are present only in one copy per genome of native strain, or per transconjugant obtained from recipient devoid of a resident element. Only two of the IMEs with high integration specificity were found in multicopy. Two copies of IME_*oriT*, an IME from *S. agalactiae*, can be specifically integrated in the conserved *nic* site of *oriT* of Tn*916* and an ICE*St3*-related element in the same native strain or transconjugant [48,63]. In addition, for SGI1 (an IME that specifically integrates in the 3′ end of *thdF* and in a secondary site), a significant fraction of transconjugants harbour tandem SGI1 arrays (up to 6 copies) [65]. It was suggested that a concatemer of several copies could be transferred to the recipient or alternatively that a single donor may repeatedly transfer a single SGI1 to a single recipient cell [65]. On the contrary, many of the IMEs with a low integration specificity can be found in 2–6 copies and in different locations of the genome. These IMEs include Tn*4453* from *C. difficile* [69], MTn*Pi1*, MTn*Pi2* and MTn*Pi3* from *P. intermedia* [44], MTn*Sag1* from *S. agalactiae* [28], Tn*4399* from *B. fragilis* [53] and Tn*4555* from *Bacteroides vulgatus* [49].

Most of the few IMEs identified before 2000 have a low specificity of integration and were thus initially classified as mobilizable transposons although all encode a serine or tyrosine recombinase rather than a DDE transposase [33,70]. However, a very large majority of the various IMEs identified later were found to have a high specificity of integration. Taken as a whole, the data obtained on IMEs encoding serine and tyrosine integrases (generally with a high specificity of integration but sometimes a low specificity, or with secondary sites) suggest that the distinction between mobilizable transposons and IMEs is not relevant. This is reminiscent of the distinction between conjugative transposons and ICEs that was extensively discussed by Burrus et al. in 2002 [13]. Thus, IMEs were defined as elements that are able to excise, use the conjugative apparatus encoded by another MGE to perform its own transfer and integrate in a replicon of the recipient cell, regardless of their specificity or mechanism of integration [12].

### 2.2. Maintenance of Excised IMEs: An Unexplored World

It has been originally assumed that, once ICEs and IMEs are integrated into the chromosome of their host, they are exclusively inherited as a part of chromosome during cell division [23]. According to this assumption, if the cell division occurs while the element is excised, the element would inevitably be lost in one of the daughter cells. Recent evidences on various models have now clearly demonstrated that ICEs use multiple strategies to ensure the transient maintenance of the excised element in the daughter cells and/or to leverage maintenance in cell populations (for a review see [20]). Even though the strategies used by IMEs to maintain as an extra-chromosomal form have not been described yet, the gene content of various IMEs and experimental data obtained for the IME SGI1 suggest that at least some of them use strategies that are similar to those of ICEs.

**Figure 2:**
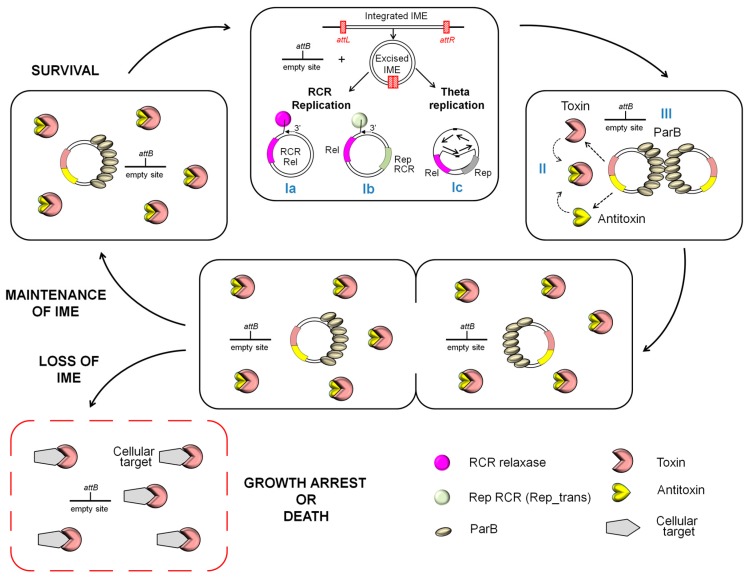
IME-encoded factors that contribute to IME maintenance in the cell during cell division: I Replication factors allowing rolling circle replication (RCR) or theta replication; II, toxin-antitoxin systems; III, partition protein ParB.

Hence, a growing body of evidence suggests that transient replication is a common, if not universal, mechanism of maintenance of excised ICEs. Indeed, the maintenance of excised ICEs Tn*GBS1* and Tn*GBS2* from *S. agalactiae*, involves replication via two different theta dedicated replication systems, independent of the conjugative relaxase [71]. In the same way, although the replication of excised IMEs has never been studied, it should be noticed that, besides their relaxase, various IMEs encode proteins that are related to factors involved in the initiation of the replication of plasmids or phages and could be involved in maintenance of the IME after excision (Figure 2). These proteins are homologous to (i) theta replication initiators belonging to the Rep_3 superfamily (SGI1 and BcenGI2); (ii) replisome organizers and DnaC that are involved in initiation of the theta replication of various prophages (Tn*6104* and various IMEs from *Streptococci*); (iii) RepA and RepC involved in theta plasmid replication (GISul2, IncP islands, *tet*(O) fragment); or (iv) RCR initiators belonging to the Rep_Trans family (various IMEs from *Streptococci* such as IME*Sag-rpsI* and IME_*Sga2069_rpmE*) (Table 1). Furthermore, many IMEs from *Streptococci* (Table 1, [26]), which do not encode such replication proteins, encode non-canonical putative relaxases that might be used for both conjugative transfer and maintenance of the excised element (Figure 2). Indeed, these non-canonical relaxases are related to RCR initiators that are involved in the replication of many small plasmids from Firmicutes (Rep_Trans/MobT, Rep_2) or of phages (Viral_Rep, PHA00330). From this point of view, it should be noticed that Tn*916* and ICE*Bs1*, two ICEs encoding non-canonical MobT relaxases, use their MobT not only as a relaxase involved in the initiation of transfer and concomitant replication but also as a RCR replication initiator involved in the maintenance of the excised ICE [72,73].

ICEs belonging to the SXT/R391 family further reduce the chance of losing their excised form by deploying active partition systems related to those of plasmids [74]. It could also be the case of some IMEs from *Streptococci*, such as the *tet*(O) fragment, that encode proteins related to ParB, a protein involved in the active partition of plasmids (Table 1 and Figure 2). ICEs often encode addiction systems such as toxin-antitoxin (TA) [21] or type II restriction–modification (RM) [75] that can trigger post-segregational killing of daughter cells that have lost the element [76]. Sequence analyses revealed complete TA or type II RM systems that possibly act as addiction system in various IMEs from Proteobacteria, Firmicutes and Actinobacteria (Table 1 and Figure 2). Only one, SGI1 from *S. enterica*, was recently studied in this perspective. SGI1, that is known to be highly stable once acquired [77], encodes a putative replication initiator and the SgiAT toxin-antitoxin system. The deletion of *sgiAT* genes does not reduce the stability of SGI1 in the absence of its helper elements, the IncA/C plasmids [78]. This correlates with the complete absence of excision of SGI1 in the absence of the helper plasmid. In the presence of IncA/C plasmids that induce the SGI1 excision, the deletion of *sgiAT* leads to a high instability and loss of the IME [78]. This indicates that SgiAT triggers post-segregational killing of daughter cells having lost the excised element and therefore probably contributes to the maintenance of the IME in bacterial population.

### 2.3. Impact of Other Mobile Genetic Elements on IME Maintenance

Although IMEs are autonomous for their maintenance, recent studies of various IMEs suggest that the interaction of IMEs with other MGEs can have profound effects on integration and excision of IMES as well as on maintenance of excised IMEs. The MGEs that can affect IME maintenance include not only helper elements but also elements that integrate in tandem with IMEs.

The impact of the presence of a helper element on the excision was studied for IMEs mobilized by the ICE CTnDOT and its relatives (NBU1 from *Bacteroides*), by IncA/C plasmids (SGI1, MGI*Vmi* and MGI*VchHai6* that belong to three different families of IMEs from Gammaproteobacteria) and by ICEs belonging to SXT/R391 family (MGI*Vfl*Ind1 from *Vibrio*). All these IMEs are able to integrate in the chromosome of their natural hosts in the absence of the helper elements. However, none of them is able to excise in their absence since the main regulators of the helper elements are required for the expression of the RDF genes carried by the IMEs [25,60]. For example, the SXT-encoded transcriptional activators SetCD trigger the expression of genes encoding the integrase and the RDF of MGI*Vfl*Ind1, promoting its site-specific excision [58]. Taken as a whole, these IMEs remain integrated and quiescent in the absence of the helper element but exploit the main regulatory mechanisms of their helper elements for timing their own excision, a crucial step of their transfer. It seems highly probable that most IMEs, if not all, act in a similar way since the excision of the IME in the absence of the helper element would inevitably increase the frequency of its loss and therefore would clearly be disadvantageous for IMEs. In this perspective, it should be noticed that, after growth of a strain harbouring only SGI1 (always integrated in the absence of the helper plasmid) during 351 generations without any selective pressure, 100% of the cells harbour SGI1. After a similar experiment on a strain harbouring both the IME and its helper element, only 72% of the cells harbour SGI1 [78].

Recent analyses of the IMEs encoding site-specific recombinases showed that they are frequently integrated in tandem with other elements. These latter can be functional ICEs, related or unrelated IMEs, prophages or satellite prophages but are generally decayed elements [12,26,48]. In most cases, these tandem structures would result from a three-step evolution: (i) the site-specific integration of an ICE or IME acquired by conjugation; (ii) the decay of this ICE or IME leading to a CIME (*cis*-mobilizable element, i.e., an element that has lost its integration and transfer genes but has retained *attL* and *attR* sites [12,79]); (iii) the subsequent site–specific integration of another incoming IME in the *attR* site including the end of the target gene, leading to the accretion of the IME and the CIME [12,48]. For tandems of functional elements, which encode integrases that share similar specificities but are generally distantly related, it is probable that an incoming element has integrated within the *att* site of a resident element. However, a scenario involving the acquisition by excision, conjugative transfer and integration of the whole composite element resulting from a former accretion (ICE-IME, IME1-IME2 or CIME-IME) cannot be excluded, since conjugative transfers of CIME-ICE tandems were also observed [45,80,81,82,83]. Such tandem structures can have complex excision patterns [12]. This is exemplified by a composite island including IME_*Sag2603_tRNAlys* from *S. agalactiae*, the only IME-including complex structure whose excision has been tested so far. This IME is integrated in the 3′ end of a tRNAlys gene, in tandem with a slightly decayed ICE (*attL*_ICE_-decayed ICE-chimerical *attI*-IME-*attR*_IME_-3′ end of a tRNAlys gene) [84]. These two integrated elements encode very different tyrosine integrases (<30% identity) and have very different *att* sites. Nevertheless, despite these differences, the IME excises by site-specific recombination between the chimerical *attI* site and *attR*_IME_ and the whole composite element excises by site-specific recombination between the *attL*_ICE_ and the *attR*_IME_ [45].

## 3. IMEs: Mobile Elements That Hijack the Conjugative Apparatus of Self-Transmissible Elements

Since IMEs encode only some of the functions needed for conjugation, their transfer requires the presence of a helper conjugative element in the donor strain. The mobilization of only a few IMEs has been studied so far and, in all cases, the transfer of the IME is independent of any co-transfer of the helper element [38,39,41,43,49]. The identified IMEs are not defective or decaying mobile genetic elements deriving from ICEs but are quiescent hitchhikers or parasites that await opportunities to use or hijack the conjugative apparatus of self-transmissible elements to promote their own transfer, using highly diverse strategies.

### 3.1. Canonical IMEs Encoding Their Own Relaxases but no T4SS Protein

**Figure 3:**
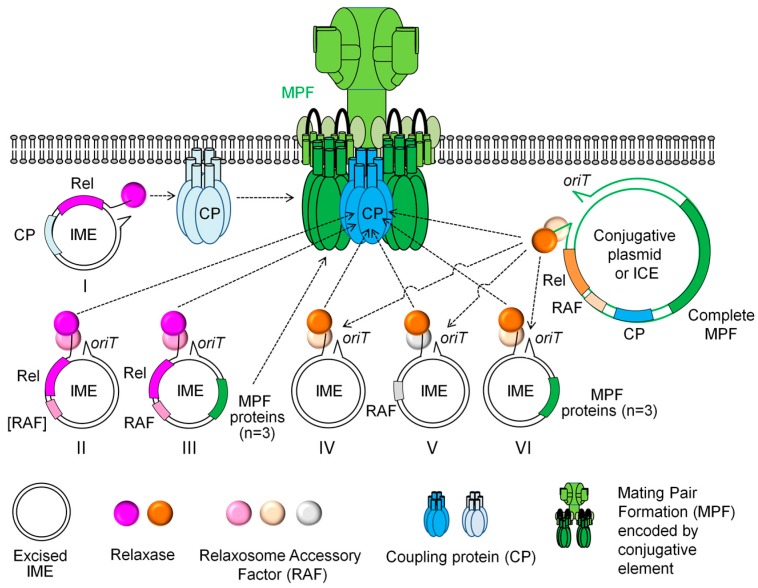
Schematic illustration of the diverse strategies of IME mobilization. Conjugative plasmids as well as integrative and conjugative elements (ICEs) encode all the proteins necessary for their autonomous transfer by conjugation, including a relaxase, relaxosome accessory factors (RAFs), a coupling protein (CP) and a Type IV secretion system (T4SS). Relaxase and RAFs are shown as colored spheres, CP as blue hexameric protein and MPF as green multimeric protein complex. IMEs can exploit conjugative elements for transfer by encoding: I, distinct relaxase and CP to recruit the MPF; II, a distinct relaxase and sometimes additional RAF to recruit the CP and MPF; III, a distinct relaxase, RAF and 3 MPF proteins to recruit the CP and T4SS; IV, only an *oriT*; V, an *oriT* and RAF to recruit the relaxase of conjugative elements; VI, an *oriT* and 3 MPF proteins. Interactions between elements are drawn as arrows with dotted lines.

Like almost all known mobilizable plasmids, various IMEs, referred here as “canonical IMEs”, encode their own canonical relaxase but do not encode CP or any MPF protein (Table 2 and Figure 3). These canonical IMEs include all the few IMEs found in the phylum Bacteroidetes, the only one found in Actinobacteria and a minority of those identified or predicted in Firmicutes. However, none of those described in Proteobacteria belongs to this category. Known canonical IMEs encode relaxases belonging to four of the six canonical families (MobP, MobQ, MobV, or MobC) but none encodes relaxases belonging to the two other canonical families (MobF or MobH). The *oriT* and the precise position of the nick introduced by the relaxases were identified for only some of canonical IMEs from Bacteroidetes and from Firmicutes [37,85,86]. Some of the canonical IMEs from Bacteroidetes and Firmicutes also encode other mobilization proteins that probably belong to their relaxosome (Table 2 and Figure 3).

The transfer by identified helper elements has been tested in their native host or using related strains or species for only a few canonical IMEs. Four IMEs from Bacteroidetes encoding a MobP relaxase (Tn*4399*, Tn*4555*, NBU1, NBU2) are mobilized by ICEs encoding a VirD4 coupling protein and a MPF_B_, i.e., a class of mating pore found only in Bacteroidetes. Although the transfer of one canonical IME (Tn*6215*) was observed in Firmicutes, no helper element of this IME was identified in its natural host [90]. However, a recombinant plasmid harbouring the mobilization module of IME*Sag-rpsI* from *S. agalactiae* HRC encoding a MobV relaxase is mobilized *in trans* by the pAMβ1 plasmid encoding a mating pore belonging to the MPF_FATA_ class [37], a class of MPFs present in Firmicutes [10]. Furthermore, once introduced in *E. coli*, various elements from Bacteroidetes (encoding MobP or MobV relaxases), Tn*4451* and its very close relative from Firmicutes (encoding MobV relaxases) and ATE-1 from the Actinobacteria (encoding MobV relaxases) are mobilized *in trans* by IncP plasmids that encode a MPF belonging to the MPF_T_ class (Table 2), a class only present in Gram negative bacteria [17]. Taken as a whole, the few available data do not allow determining the extensive network of mobilization for any of these IMEs in their native hosts.

### 3.2. Non-Canonical IMEs Devoid of Relaxases

Various non-canonical IMEs that do not encode any relaxase have recently been identified in Firmicutes and Proteobacteria. These elements are reported here as MGIs for mobile genomic islands, as proposed by Daccord et al. [27].

Two very small (1.7–2.0 kb) MGIs from Firmicutes, MTn*Sag1* [28] and its relative tIS*Cpe8* [29], are mobilizable by Tn*916*. These elements only encode a lincosamide resistance and a DDE transposase that is responsible for their excision and insertion [28]. The origins of transfer of these MGIs are probably not related to *oriT* of Tn*916*, as they are located within the resistance gene and have non-significant similarities to the *oriT* of Tn*916* (50% identity on only a 60 bp region inside of *oriT*) [28]. These data suggest that the *oriT* of MTn*Sag1* might have arisen by chance and that its recognition by the conjugative machinery of Tn*916* might only be fortuitous, as proposed by Daccord et al. [27].

A family of 17 large (18–33 kb) MGIs from various Gammaproteobacteria, mainly from *Vibrio*, encode their own tyrosine integrase and RDF but do not encode any protein sharing significant similarities with proteins involved in conjugation [27,40]. These elements carry an *oriT* that is related to those of the ICE SXT^M010^ and its relatives (highly significant 63% identity on 282 bp). One of them, MGI*Vfl*Ind1, was shown to be mobilized *in trans* by ICE*Vfl*Ind1 that belongs to the SXT family [27]. It was demonstrated that the *oriT* of the MGI and the relaxase together with the relaxasome protein MobI of the ICE are needed for the mobilization. Taken as a whole, the mobilization process can be viewed as follows: once the main regulators SetCD of the ICE induce the excision of the MGI (see the Section 2.3), the *oriT* of the MGI is recognized by the MobH relaxase encoded by the ICE and the IME is transferred as a single-stranded DNA molecule through the VirD4 CP and MPF_F_ encoded by the ICE [27]. Therefore, these MGIs appear to take advantage of the regulatory and conjugative machinery encoded by SXT-related ICEs to transfer to the recipient cells. In the same way, a family of putative MGIs that carry a sequence related to *oriT* from ICE*Kp1* (an ICE unrelated to SXT) but do not encode any protein related to conjugation proteins was identified in *Klebsiella pneumoniae* [87] (Figure 3).

Two other families of MGIs (exemplified by MGI*Vch*Hai6 and MGI*Vmi*1) recently found in Gammaproteobacteria, do not encode any protein sharing significant similarities with relaxases, CPs or MPF proteins but encode a putative relaxosome protein MobI related to that of the IncA/C plasmid family [39,41]. These plasmids mobilize *in trans* MGI*Vch*Hai6 and MGI*Vmi*1 and the main regulator of the plasmids (AcaCD) induces the expression of *mobI*. Combined to data concerning their excision, the following model for their lifecycle can be drawn [39,41]. In the absence of helper element, the MGIs remain quiescent in their integrated chromosomal state. After the entry of an IncA/C plasmid, AcaCD triggers the synthesis of the MGI RDF, leading to the excision of the IME. It also triggers the synthesis of MGI MobI that would bind to the unidentified *oriT* of the MGI and would recruit the IncA/C plasmid relaxosome including a MobH relaxase (Figure 3). The IME then transfers as a single-stranded DNA molecule through the VirD4 CP and MPF_F_ encoded by the conjugative plasmid. In the recipient cell, once the complementary strand is synthesized, the constitutive expression of the *int* gene allows site-specific integration of the MGI, regardless of the presence of the helper IncA/C plasmid.

Another family of MGIs (exemplified by SGI1, a 42 kb MGI from *Salmonella enterica*) is specifically mobilized by IncA/C conjugative plasmids [78,92]. This element and its relatives do not encode any protein related to relaxase, relaxosome components, CP or any MPF protein, except three proteins showing 40, 60, et 78% identity with TraG (a VirB6 homolog), TraH (a pilus protein) and TraN (a protein involved in mating pair stabilization), from the MPF_F_ of the IncA/C plasmids [93]. Although their expression is triggered by the master activator AcaCD of IncA/C plasmids [25], these genes are dispensable for SGI mobilization [77]. Mating experiments using combinations of deletion mutants of SGI1 and/or a helper IncA/C plasmid revealed complex interactions between these two MGEs [25]. Although SGI1 is able to use a mating apparatus encoded by the helper plasmid, the replacement of the TraN, TraG and TraH MPF subunits of the plasmid by the ones of SGI1 is necessary for the optimal mobilization process, which largely surpasses the transfer rate of the helper plasmid. The replacement of the plasmid-encoded TraG by the one of the MGI disables the IncA/C-encoded entry exclusion mechanism, i.e., the mechanism that prevents the transfer of an IncA/C plasmid to a cell that already harbors an IncA/C plasmid. Retromobilization of SGI1 is observed in mating experiments involving a strain harboring only the IME and another harbouring only the helper plasmid [94]. The retromobilization process is thought to involve first the transfer of the plasmid from the strain harbouring the plasmid to the cell harbouring only SGI1, leading to a cell harbouring both elements. Then, in these cells, the main regulator of IncA/C plasmid would induce the SGI1 excision and the SGI1 MPF subunits synthesis. These MPF subunits would replace the ones of the plasmid, disabling the plasmid exclusion system and allowing SGI1 to invade efficiently the strain harbouring only the plasmid (Figure 3).

In a similar way, although they are not related to SGI1, three adjacent genes of the putative MGI GI*Sul2* and its relatives from various Gammaproteobacteria, encode proteins related to TrbJ (VirB5), TrbK (protein exclusion entry) and TrbL (VirB6) MPF_T_ subunits from IncP1-α plasmids [36]. Like SGI1, they do not encode any other protein related to relaxase, other relaxosome proteins, CP nor other MPF subunits. Therefore, it seems likely that GI*Sul2* not only uses the MobP relaxase, the relaxosome and CP proteins of the helper plasmids but also reshapes their MPF_T_ to promote its own retrotransfer, allowing this MGI to invade strains harbouring IncP1-α plasmids and perhaps other IncP plasmids [36] (Figure 3).

### 3.3. Non-Canonical IMEs Encoding Their Own Canonical Relaxase and Some Proteins of the T4SS

Like various canonical IMEs, the putative IncP island from the Betaproteobacterium *Burkholderia glumae* 5091 and its relatives found in Gammaproteobacteria and Alphaproteobacteria were recently found to encode their own MobP relaxase related to those of the IncP conjugative plasmids [89]. The IncP island also encodes proteins related to TraJ and TraK relaxosome proteins of IncP plasmids. However, unlike canonical IMEs and like the MGI GI*Sul2*, besides these relaxosome proteins, the IncP island encodes two MPF proteins TrbJ (VirB5) and TrbL (VirB6) related to those of IncP plasmids. Furthermore, although it has not been described by Yoshii et al. [89], our reanalysis of the intergenic *trbJ*-*trbL* sequence reveals a small gene encoding a 69 aa protein. The comparison of the structure of IncP island with the one of the IncP plasmid RP4 suggests that this small protein could be an exclusion entry protein. Indeed, in this plasmid, the gene *trbK* located between *trbJ* and *trbL* encodes a small 69 aa protein that is the only protein involved in the exclusion entry of RP4 plasmid [95,96]. Therefore, these data suggest that the IncP island, like canonical IMEs, encodes its own relaxosome and, like the MGI SGI1, subverts the MPF system of its helper elements, probably IncP plasmids, to promote its own transfer and the invasion of cells harbouring IncP plasmids by retrotransfer (Figure 3).

Furthermore, two putative IMEs of *Streptococcus* were recently found to encode their own VirD4 CP and a MobC relaxase [26]. This is reminiscent of plasmids, since the few known mobilizable plasmids that encode their own VirD4 CP also encode a MobC relaxase [7]. These elements probably replace the CP from the T4SS of the helper conjugative element by their own CP to promote their transfer (Figure 3).

### 3.4. Non-Canonical IMEs Encoding a Non-Canonical Relaxase and/or CP

The ssDNA transfer of conjugative or mobilizable elements has been, for a long time, thought to involve canonical relaxases (i.e., MobC, MobF, MobH, MobP, MobQ, or MobV) and canonical CPs (VirD4). However, recent analyses of conjugative elements from Firmicutes revealed that many of them use non-canonical relaxases. The conjugative plasmid pCW3 and its relatives use a tyrosine recombinase as a relaxase [97]. Furthermore, the ICEs belonging to the Tn*916*/ICE*Bs1*/ICE*St3* family use MobT relaxases that are related to Rep_Trans initiators, i.e., proteins involved in the maintenance of many small plasmids of Firmicutes [17]. In addition to their relaxase function, the MobT/Rep_Trans proteins of Tn*916* and ICE*Bs1* also catalyse the initiation of RCR needed for the maintenance of the excised ICEs [72,73]. Furthermore, “RCR initiators” belonging to another family (Rep_1) are also involved not only in the maintenance of three plasmids from Firmicutes but also in their mobilization by ICE*Bs1* [98]. Therefore, the classical distinction between RCR initiators and relaxases is probably not relevant.

Unexpectedly, an extensive analysis, very recently performed on 124 genomes of *Streptococcus*, revealed that 118 among the 144 IMEs detected in these genomes encode a putative non-canonical relaxase related to rolling circle replication initiators [26]. Among these 118 IMEs, 45 encode RCR initiators/relaxases belonging to the MobT/Rep_Trans family. The other 73 IMEs encode putative relaxases related to other RCR initiator families [PF01719/Rep_2 (35 elements), PHA00330 (21 elements), PF01719/Rep_2 associated with a helicase domain (15 elements) and PF02407/Viral-Rep (2 elements)]. Among these 118 IMEs, 35 do not encode any CP. Unexpectedly, the 83 other IMEs encoding a RCR initiator-related relaxase (24 with MobT, 34 with PF01719, 19 with PHA00330, 4 PF01719 associated with a helicase domain, 2 with PF02407) also encode a non-canonical CP (called TcpA). TcpA CPs are distantly related to FtsK, a protein involved in dsDNA translocation during cell division and to TraB, a protein involved in dsDNA conjugative systems from Actinobacteria. It should be mentioned that all the very few known events of mobilization *in trans* of plasmids involving a RCR initiator-related relaxase also involve a TcpA CP encoded by the helper ICE [98,99,100]. This suggests that the putative IMEs encoding relaxases related to RCR initiators can only hijack conjugative elements that encode TcpA. Surprisingly, within these IMEs, closely related relaxases can be associated, or not, with a TcpA CP, or can be associated with distantly related TcpA CPs. Furthermore, the phylogenetic analyses of the relaxases and CPs reveal losses, acquisitions and replacements of TcpA genes between IMEs. Therefore, it seems probable, that, like MPF subunits encoded by the MGI SGI1, the CPs encoded by these IMES would be dispensable for their mobilization but that the replacement of the CP of the helper conjugative element by the one of the IME would promote their efficient mobilization, probably at the expense of the helper element.

### 3.5. IMEs: Harmless Hitchhikers or Harmful Pirates of Conjugative Elements?

Recent works showed that at least some IMEs not only need conjugative elements to promote their own transfer but also influence the transfer or stability of host or helper conjugative elements.

**Figure 4:**
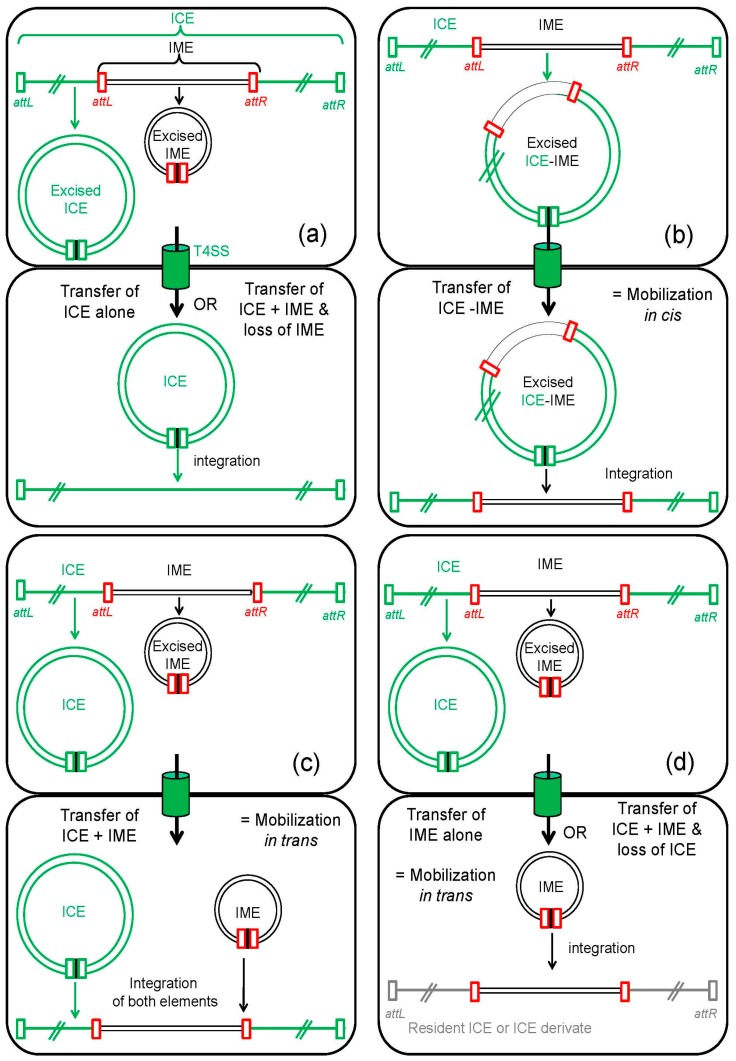
Schematic representation of the different scenarios of IME mobilization in the case of an IME integrated in an ICE. The IME can be integrated in a conserved gene of the ICE or in *oriT* of the ICE. (**a**) The IME and ICE excise. Then, the ICE transfers alone, or both elements transfer (through the T4SS encoded by the ICE appearing as a green cylinder on the figure) but the ICE is lost; (**b**) ICE and IME excise and transfer together as a composite ICE-IME element. Then, the composite element integrates in the chromosome of the recipient cell (mobilization *in cis*); (**c**) The IME and the ICE can excise and separately transfer in the recipient cell (mobilization *in trans*). If the IME is integrated in a gene essential for the transfer (for example the CP gene), this prior IME excision is required for ICE transfer and mobilization *in trans* of the IME; (**d**) IME can excise and transfer alone (also by mobilization *in trans*) or ICE can be lost after transfer without loss of the IME. The IME integrates in a resident ICE or in a derivate of ICE. Boundaries of the elements (attachment sites *att*) are shown as rectangles: green for the ICE that can mobilize the IME, red for the IME and grey for the resident ICE/derivate of ICE.

Various ICEs from Firmicutes, belonging to the Tn*5252* superfamily, carry 1 to 3 different IMEs specifically integrated in conserved genes that are therefore disrupted by the IME insertion [12,26,32,101,102]. Two of the three targeted genes encode unknown proteins related to Maff2 proteases or to SNF2 helicases [26] (Table 1). Only one of these ICEs, ICE*Sp2905*, was tested for transfer [30]. This ICE carries two different IMEs, one (IME*Sp2907*) integrated in the gene encoding a Maff2-related protein and the other (*tet*(O) fragment) integrated in a gene encoding a helicase-related protein. Three types of transconjugants were recovered from ICE*Sp2905* transfer assays. One corresponds to the transfer of the IME*Sp2907* element alone, i.e., a mobilization *in trans* of the IME and its insertion in a resident ICE (Figure 4). Another corresponds to the transfer of an ICE devoid of IME*Sp2907* but carrying the *tet*(O) fragment, suggesting that IME*Sp2907* excised from ICE*Sp2905* and was subsequently lost, or did not transfer (Figure 4). The last one corresponds to the transfer of the whole ICE carrying the two IMEs. At first sight, this suggests that the disrupted genes are not required for ICE transfer and that IMEs are mobilized *in cis* by ICE*Sp2905*. However, it should be noticed that the disrupted genes are conserved in all related ICEs and are located next to genes involved in conjugation. Furthermore, besides IMEs integrated in Maff2 or SNF2 encoding genes, ICEs belonging to the Tn*5252* superfamily can also carry up to two IMEs (e.g. IME_*Sco1050_traG_site1* and IME_*Sco1050_traG_site2*, Table 1) integrated in two different sites of the gene encoding the VirD4 CP, a protein essential for transfer [26]. Although none of these ICEs was tested for transfer, insertion of IMEs within genes required for conjugation would abolish the ICE transfer and therefore the mobilization of the IME by the host ICE. However, as it has been recently proposed [26], the induction of ICE transfer could provoke the excision of the IME, leading to a fully functional conjugation module. This would allow not only ICE transfer but also IME mobilization if this latter element uses the MPF of the ICE to transfer and integrates in the ICE in the transconjugant (Figure 4). According to this hypothesis, the IME would be a harmless hitchhiker that uses the ICE both as a site of integration and as a transfer engine. A similar mobilization mechanism can also be proposed for IMEs that are site-specifically integrated in the putative *oriT* from ICEs belonging to Tn*916* and ICE*St3* families that are found in *Streptococci* [48,63].

The impact of IMEs on the transfer and/or the stability of the helper co-resident elements that mobilize them *in trans* has only been studied for SGI1 family. This family of IMEs is mobilized only by the conjugative plasmids belonging to the IncA/C incompatibility group [92]. While all these IMEs and plasmids encode multiple antibiotic resistances [78], surprisingly, SGI1-related element and IncA/C plasmids are never found together in clinical multidrug resistant *Salmonella* isolates*.* Moreover, after cultivation of a strain harbouring pRMH760 (an IncA/C plasmid) and SGI1-I during 110 generations without any selective pressure, all cells have retained the IME, whilst <1% have retained the plasmid. On the contrary, no plasmid loss was observed in the absence of the IME [103]. Similar experiments performed with another IncA/C and/or two other SGI1-related elements also resulted in the loss of the helper plasmid. Only one IME, SGI1-K, does not destabilize the helper plasmid. Unlike other SGI1-related elements, SGI1-K does not encode the two putative proteins S006 and S007 related to AcaCD, the main regulator of IncA/C plasmids. This suggests that S006 and S007 from SGI1 elements would be involved in the instability of the plasmid [103]. The transfer frequencies of the IME, of the helper plasmid and co-transfer from a donor strain harbouring both a SGI1-related element and an IncA/C plasmid are variable, depending on the IMEs, plasmids and strains used for the conjugation experiments. It should be noticed that these co-transfers are always less frequent that the transfer of the IME alone or of the helper element alone when using strains harbouring both the IME and the helper plasmid [103]. For some IMEs, helpers and/or bacterial strains, this co-transfer does not occur or is very infrequent although the frequencies of transfer of helper plasmids alone and/or IMEs alone from strains harbouring both elements are high [38,41]. Furthermore, the frequency of transfer of the IncA/C plasmid pRMH760 was 1000–5000 fold lower when the donor carries both pRMH760 and SGI1-related elements than when the donor carries only pRMH760, indicating that the SGI1 element suppresses the transfer of the helper plasmid [103]. Taken as a whole, the recent data concerning the stability and transfer of the helper plasmid in the presence of the IME show that SGI1 does not only use the conjugative apparatus of the helper elements but is a harmful pirate. This IME is able to invade by retrotransfer the cells harbouring only the IncA/C conjugative plasmids (see previous sections); then it suppresses the plasmid transfer and leads to the plasmid loss in the cell. This would lead to the displacement of IncA/C plasmids by SGI1 in cell populations carrying IncA/C plasmids upon contact with cells carrying only SGI1 [104].

Apart from SGI1, the impact of the IME on the transfer and/or stability of helper co-resident element has not been studied. However, the co-transfer of MGI*Vmi*1, an IME unrelated to SGI1 and of its helper elements, the conjugative plasmids IncA/C, does not occur, suggesting that the IME affects the transfer of the helper element or its maintenance in the transconjugant [39]. In strains harbouring other IMEs, unrelated to SGI1 and to MGI*Vmi*1 and their helper element, the co-transfer of both elements is generally less frequent that the transfer of each element alone. Nevertheless, unlike SGI1 and MGI*Vmi*1, the co-transfer is generally significant [41,43,49], suggesting that the IME and its helper element are compatible.

## 4. Moving with IMEs: Their Cargo Genes

The evolutionary success of IMEs in a population depends, not only on their ability to invade novel hosts and to be transmitted to the host descent but also on their impact on the host fitness. This impact has never been studied so far but should not be neglected since all IMEs, except Tn*5520*, harbour cargo genes that can contribute to the fitness of their host. Even the smaller one, MTn*Sag1* (1.7 kb) encodes lincomycin resistance [28]. The presence of a variable array of cargo genes is the main cause of the variability of the size of IMEs. Most IMEs are between 5 and 15 kb long. The longest IMEs hardly exceed 50 kb, even when they carry other integrated MGEs, such as an integron (Table 3).

### 4.1. IMEs: A Reservoir of Antibiotic Resistance Genes

A large proportion of identified IMEs carry antibiotic resistance genes. The characterization of their potential spreading (between pathogenic or from commensal strains) is therefore a major challenge. Tetracycline resistance is encoded by various IMEs, such as ATE-1 carrying *tet*(W) in various strains of the Actinobacterium *Trueperella pyogenes* [34] (Table 3) or Tn*6031* that carries the *tet*(X) gene in the Bacteroidetes *Sphingobacterium* sp. PM2-P1-29 [46]. Some *Streptococcus pyogenes* strains resistant to tetracycline and erythromycin harbour an ICE carrying two IMEs [31]. One of them is the *tet*(O) fragment and the second one (IME*Sp2907*) carries the erythromycin resistance gene *erm* (TR). IME*Sp2907* was also identified (99% DNA identity) in several strains of *S. agalactiae* [101]. Lincosamide resistance genes are also frequent on IMEs. *C. perfringens* strains, resistant to lincomycin and susceptible to erythromycin, host tIS*Cpe8*, an IME carrying the *lnuP* gene [29]. This element is related to MTn*Sag1* (IME carrying the *lnu*(C) gene conferring resistance to lincomycin) previously characterized in *S. agalactiae* [28]. Other IMEs (IME_*oriT*) found in *S. agalactiae* carry the *lsa*(C) gene conferring lincosamide, streptogramin A and pleuromutilin resistances [48,63]. Furthermore, two *Bacteroides* clinical isolates harbour the IME NBU2, carrying the *linAN2* resistance gene [47]. Genes conferring resistance to other families of antibiotics are also found on IMEs. This is the case of an IME related to tIS*Cpe8* found in *S. suis* that carries a chloramphenicol acetyl transferase *cat* gene [29]. An element of *C. difficile*, Tn*4453*, carries the chloramphenicol resistance gene *catD* [69]. This latter IME is closely related to Tn*4451* from *C. perfringens* that carry a *catP* gene [54]. A gene (*cfxA*) encoding an extended spectrum β-lactamase is also present on Tn*4555* [49] in various *Bacteroides* species [109].

There are also various examples of IMEs associated with multidrug resistance (Table 3). SGI1, firstly found in *S. enterica* Typhimurium DT104 [38], carries an impressive set of antibiotic resistance genes (Table 3), due to the presence of a class I integron (In104) inserted in the IME backbone. Since this first identification in *Salmonella*, many variants of SGI1 have been described. To date, 43 variants have been identified [110], not only in several serovars of *Salmonella* but also in other Enterobacteria. The *Proteus mirabilis* SGI-V variant ensures the carriage of 6 antibiotic resistance genes [107] and the SGI-L variant brings 5 antibiotic resistance genes to *Morganella morganii* [108,111]. Lately, other IMEs unrelated to SGI1, such as MGI*Vch*Hai6 from *Vibrio cholerae*, were also found to carry an In104-related integron encoding various antibiotic resistances [39]. Kasugamycin (KSM) is an antibiotic used in agriculture to protect rice against *B. glumae* or *Acidovorax*. Emergence of KSM-resistant strains is linked to a novel resistance gene carried by an integron cassette that is inserted in an IncP island [89,112]. The IncP family of IMEs was also identified in several species of *Brucella* [35]. With over 100 gene cassettes described, mainly encoding antibiotic resistance, integrons appear as efficient systems of gene capture. Therefore, their integration in IMEs would facilitate their transport and would potentiate the dissemination of antibiotic resistance genes.

It should be emphasized that some antibiotic resistances, which were initially attributed to plasmids or ICEs, are actually encoded by IMEs integrated into these MGEs. Thus, the ICE CTnDOT from *Bacteroides* was thought to encode not only a tetracycline resistance, like its close relative CTnREL but also an erythromycin resistance [113]. This latter is now known to be encoded by an IME derivate carried by the ICE, the *ermF* region [106]. The *C. perfringens* plasmid pIP401 was studied for its ability to transfer a chloramphenicol resistance [114] while the real carrier of the *catP* gene is the IME Tn*4451* [69] held by the plasmid. This situation is comparable to that of GI*sul2*, an IME inserted in pIP40a, an IncA/C plasmid [36].

The IME*Sp2907* [30] and IME_*oriT* with the *lsa*(C) gene [63] can all be transferred with/or without the ICE that carry them. Joined to the current lack of knowledge about IMEs, this suggests that IMEs could be more involved in the spreading of antibiotic resistance genes than initially thought. For instance, it was recently proposed that the various ARI-B resistance islands seen in many IncA/C plasmids arose from the IME GI*sul2* integrated into the plasmid backbone [36].

### 4.2. Other Putative Functions Encoded by IMEs

Besides antibiotic resistance, the cargo genes carried by IMEs confer a very large array of functions that could be advantageous for the bacterial host (Table 3). The recent sequence analysis of almost 200 IMEs, integrated in the tRNAlys CTT gene [48] or *rpsI* gene [37] of *S. agalactiae* revealed some putative adaptive functions such as intracellular protease, resistance to arsenic and to mercury [48,84]. Comparative genome analysis of the Bacteroidetes *P. intermedia* clinical strain OMA14 with strain 17 led to identification of fifteen IMEs and IME derivates [44]. Several of them encode ABC transporter components or a subtilase-like protease or a LuxR family transcriptional regulator that could affect their host behaviour.

Looking for IMEs in different genomes of various Proteobacteria led to the characterization of 11 related IMEs [40] carrying type I, II, or III RM. RM systems were first described as bacterial innate immune systems allowing protection against foreign unmethylated DNA [76]. Unmethylated incoming DNA will be degraded by restriction endonucleases produced by the cell while the genome of the host (self) remains protected due to methylation by the cognate methyltransferase. RM systems carried by IMEs could protect their host from invasion by other genetic elements and thus play a role in “cellular defence” as described for other bacteria [115]. However, the role of RM systems in bacteria appears wider than only defence. They maintain heterogeneity of a bacterial population and are involved in adaptation of bacteria to environmental changes [116]. RM systems also turn out to be themselves selfish mobile elements and can participate to bacterial genome evolution [117]. Although type II RM systems encoded by IMEs (e.g., IME MGI*Vch*Moz6) can be involved in the stability of the IME, they can also be engaged in defence against incoming DNA, especially bacteriophages (e.g., type I RM for IME MGI*Vch*Hai6; type III RM for IME MGI*Vmi*1).

Many genes carried by IMEs are homologous to toxin-antitoxin systems (TA) that could be involved either in the IME maintenance or in cellular functions. Chromosomal TA systems control the rate of intracellular metabolisms to regulate cell growth and death under stress conditions [118]. The IME MGI*Vch*USA1, identified in *V. cholerae*, encodes two TA systems related to the chromosomal HipA-B system [27]. HipB is a DNA binding protein that was demonstrated to repress multiple promoters in *E. coli* and HipA enhances the repressor activity of HipB [119]. Another role played by HipA is to inhibit global protein synthesis and drive cells into dormancy in a stochastic way [120]. This subpopulation of phenotypic variants, called persisters, was demonstrated to be responsible for the inability of antibiotics to eradicate infection (bacterial multidrug tolerance due to their ability to resume growth after antibiotic removal) [121]. Considering the large occurrence of RM and TA systems on IMEs [27,36,37,41,61,105,108], further characterizations are needed.

The same is true for the putative antibacterial peptide production (lantibiotic) due to Tn*6104* [32] or microcin due to GIE492 [87], whose functions have not been studied yet. Other putative function of cargo genes carried by IMEs are arsenate and arsenite resistance, encoded by GI*sul2* [36], or mercury resistance encoded by IME_*18RS21_oriT* [48] and MGI*Vch*Hai6 [39].

Except for very small IMEs such as tIS*Cpe8* [29], MTn*Sag1* [28] or Tn*5520* [50], many of the genes carried by IMEs have no predicted function. As well as ICEs [122], IMEs carry a large part of genes with unknown or unannotated functions. It seems highly probable that besides unidentified genes involved in their transfer or maintenance, many of these genes could contribute to the adaptation of their bacterial host.

## 5. Evolution of IMEs

As for other types of MGEs, module acquisition or exchange play a key role in evolution of IMEs. This evolution can lead to exchanges of all types of modules between IMEs. For example, the MGIs SGI1 and MGI*VchHai6* share very closely related integrons and more distantly related integration/excision modules (integrases and RDFs sharing 67% identity and 37% identity), whereas their mobilization modules are unrelated [39]. In the same way, the recent comparison of sequences and phylogenetic analyses of integrases and mobilization modules of 144 IMEs from streptococcal genomes reveals many discrepancies that are probably due to multiple replacements of integration/excision or mobilization modules between IMEs [26]. This study also reveals acquisitions or replacements of CP gene between IMEs within the mobilization module.

Sequences analyses suggest that IMEs also exchange adaptation or integration/excision modules with other types of MGEs, especially with the conjugative elements. For example, the integrons carried by the MGI SGI1 and the conjugative plasmid pSN254b from *Aeromonas salmonicida* are very closely related [123]. Furthermore, the phylogenies of integrases of the numerous IMEs and ICEs identified in 52 *Streptococcus* genomes indicate that many of these elements share related serine or tyrosine integrases and suggest some exchanges of integration/excision modules between ICEs and IMEs [26].

The mobilization modules (or part of these modules) of various IMEs or putative IMEs (SGI1, MGI*Vch*Hai6, MGI*Vfl*Ind1, MGIV*m*i1, GISul2, IncP island and their relatives) are related to some regions of the conjugation of their helper conjugative elements (plasmids or ICEs) (see Section 3.2 and Section 3.3). This suggests that these IMEs could have initially acquired their mobilization module from conjugative plasmids or from ICEs. However, the mobilization modules of other IMEs are unrelated or very distantly related to those of their helper elements. For example, IME*Sag-rpsI* that encodes a MobV relaxase is mobilized *in trans* by the plasmid pAMβ1 that encodes a MobQ relaxase [37]. In the same way, all the relaxases and CPs from the 144 IMEs identified in 52 genomes of streptococci are distantly related or unrelated to those of the 131 ICEs (or slightly decayed ICEs) identified in these genomes [26]. Furthermore, the *oriT* of MTn*Sag1* that is the only sequence of this MGI involved in its mobilization is probably not related to *oriT* of its helper ICE, Tn*916* (see Section 3.2).

The exchange or acquisition of modules could be favoured by the presence of transposons or ISs carried by IMEs and other MGEs. They could also be favoured by the integration of IMEs in conjugative plasmids or ICEs, or by the integration of the incoming ICEs and IMEs in the *att* sites flanking resident ICEs or IMEs that share identical specificity of integration (see previous sections and [12]).

## 6. IMEs: An Obscure World to Explore

Despite their intracellular and intercellular mobility, IMEs are very difficult to detect by conjugation, which explains why they are so little studied. Indeed, most of them integrate in specific sites avoiding the disruption of the target gene. Furthermore, many of them do not encode functions that could be useful to select the transconjugants. Finally, their transfer requires the presence of a helper element that may be incompatible with the IME, as for SGI1. This explains why very few IMEs have been shown to be mobilizable *in trans* by conjugative elements (Table 2). The detection of IMEs by *in silico* analysis of genomes is also difficult. Due to their modular evolution, distantly or closely related modules can be shared by IMEs and by other types of MGEs. IMEs are characterized by an association of an integration/excision module with a mobilization module. The integration/excision modules of IMEs encode integrases or transposases that are related to those of other classes of integrated elements such as transposons, ICEs, satellite prophages or prophages and therefore their assignation to IMEs is not straightforward. Moreover, IMEs differ from all other types of integrated MGEs by the presence of a mobilization module that is difficult to detect. Indeed, the mobilization module of an IME encodes no, only one or very few conjugation proteins involved in various steps of the transfer (relaxase, relaxosome proteins, CP, VirB6, VirB8, exclusion proteins) that are related (or not) to the proteins of the helper element. Most IMEs possess other modules that can be involved in their maintenance (e.g., replication or toxin-antitoxin) or that can contribute to the adaptation of their bacterial host (e.g., resistances or metabolic function). However, all these accessory modules can also be found in other types of integrated MGEs such as ICEs or transposons. Consequently, IMEs found by sequence analysis were frequently confused with other classes of MGEs (for a review see Bellanger et al. [12]) or with decayed elements deriving of ICEs. Taken as a whole, careful sequence analysis is needed to detect IMEs within bacterial genomes.

Due to the difficulty of their detection, very few data about prevalence of IMEs were recently obtained by genome analyses. Only two comprehensive searches of all IMEs encoding a relaxase were performed on a significant number of genomes from a taxon. The first one, based on BLAST searches using as queries the relaxases and integrases of IMEs and ICEs from Firmicutes, was performed on the 8 complete or incomplete available genomes of *S. agalactiae* and revealed 9 IMEs [84]. The second one, also based on BLAST searches but using a more complete panel of integrases and relaxases, was performed on 124 genomes of 27 streptococcal species. This very recent analysis revealed 144 IMEs, which would belong to 39 distinct families according to their integrase, relaxase and CP content [26]. It also showed that the number of IMEs highly varies from species to species (from 0.1 to 4 IMEs per strain for a given species) and suggested a positive correlation between IME and ICE contents. Another analysis finds 6 ICEs (4 complete and 2 decayed) and 15 IMEs (10 complete and 5 decayed) in the genome of a strain of *P. intermedia* [44]. To our knowledge, only three comprehensive searches for putative IMEs related to a studied IME encoding a relaxase were performed on a significant number of genomes from a taxon. They showed that three families of IMEs have a high prevalence either in *S. agalactiae* [37,48] or in larger set of species from Firmicutes [124]. None was performed in another bacterial division. Furthermore, an exhaustive search for conjugation modules based on hidden Markov model profiles deduced from known relaxases (including canonical ones and MobT), CPs and MPFs, i.e., essentially conjugation modules of proteobacterial plasmids, was performed on 1124 archaeal and bacterial genomes. It detects 402 chromosomal relaxase genes that are not associated with CPs or MPFs [1] and therefore are probably carried by IMEs. This analysis also suggests that IMEs are the most frequent class of elements that transfer by conjugation. The authors of this study also suggested that the strong bias in the initial dataset for proteobacterial plasmids did not allow the detection of very distant or unrelated genes, precluding the detection of many elements in other bacterial divisions and archaea. Furthermore, comprehensive searches of MGIs (IMEs that do not encode their relaxases) have never been performed. However, searches for putative MGIs related to a studied MGI suggest that at least some of them are widespread in various species or genera of Proteobacteria [39,40,87,125]. Numerous genomic islands possess a complete integration/excision module but do not encode any reported conjugation protein (see for examples [126,127,128]). These elements could correspond to decayed MGEs, or more probably to functional but poorly known classes of MGEs such as satellite prophages [129] or IMEs. It seems probable that many of them correspond to: (i) IMEs that do not encode any conjugation protein but harbour an *oriT*; (ii) IMEs that do not encode any relaxase but encode some other conjugation proteins that have escaped to detection; (iii) IMEs encoding a relaxase belonging to a not yet characterized family. Furthermore, it should also be noticed that at least some tISs encoding a DDE transposase (i.e., insertion sequences with passenger genes) are IMEs, as it was found for MTn*Sag1* (also named tIS*Sag10*) [130] or tIS*Cpe8* [29]. Altogether, these data suggest that IMEs probably have a very high prevalence in genomes. This class of elements could even be the most widespread of all classes of MGEs that are able to transfer by conjugation, even though it is by far the least known and the least studied.

## 7. Concluding Remarks

IMEs are MGEs that encode their own excision and integration and are able to hijack or subvert the mating apparatus of conjugative elements (plasmids or ICEs) to promote their intercellular transfer. Although the IMEs are the least known of all the elements transferring by conjugation, the analyses of genomes strongly suggest that these mobile elements are even more widespread than ICEs. The current state of knowledge on these elements reveals a complex lifestyle. They display a high diversity of integration sites linked to their integration toolkit (tyrosine or serine recombinase with high or low specificity, DDE transposase). Besides integration, they may deploy different strategies to maintain in the cell after their excision (replication, addiction systems, partition). Diverse schemes of IME mobilization can be drawn, depending on their genetic localization (alone or in tandem with ICEs, in the chromosome or inside a conjugative element, targeting or not an essential gene of conjugative elements). Mobilization schemes also rely on their characteristics (IMEs devoid of relaxase, encoding or not relaxosome accessory factor(s), elements encoding a non-canonical CP and/or a non-canonical relaxase, elements encoding some T4SS proteins that reshape the mating apparatus of conjugative elements and/or disable the exclusion surface of the helper element to promote their own transfer). Their evolutionary success also depends on the various cargo genes that are carried by IMEs and could enhance the fitness of the host cell. It is particularly striking that a large part of IMEs found in commensals and pathogens vehicle antibiotic resistance genes. This substantially relies on other genetic elements, in specific transposons and integrons that were found to be inserted in various IMEs. In particular, IMEs appear as a likely reservoir of antibiotic resistance genes and, as such, should not be neglected in the strategies to fight this world growing issue. It also becomes apparent that the interactions of IMEs with their host cell and with the conjugative elements that mobilize them are complex and diverse. Upcoming scientific advances on the structural characterization of the conjugation machinery and on the mechanisms of regulation of transfer of the helper conjugative elements will pave the way to understand how IMEs pirate them. Many research fields remain to be explored to capture a full image of the capacity of horizontal gene transfer and of the impact on bacterial genome evolution of IMEs.

## Figures and Tables

**Table 1:** Maintenance of Integrative Mobilizable Elements (IMEs) and putative IMEs.

IME ^a^	Species (Division) ^b^	Int ^c^	Integration Site ^d^	Putative Maintenance Genes ^e^	Reference
MTn*Sag1*	*Streptococcus agalactiae* (fir.)	DDE	AT-rich regions	None	[28]
tIS*Cpe8*	*Clostridium perfringens* (fir.)	DDE	AT-rich regions	None	[29]
*IME_SpnAP200_rumA*	*Streptococcus pneumoniae* (fir.)	Ser	Internal site of *rumA* (23S rRNA methyltransferase)	Replisome organizer	[26]
IME_*ScoC232_maff2_site1*^f^	*Streptococcus constellatus* (fir.)	Ser	Internal site S1 of a gene (Maff2-related) from Tn*5252*-related ICEs	Replisome organizer, DnaC	[26]
IME*Sp2907* ^f^	*Streptococcus pyogenes* (fir.)	Ser	Internal site S2 of a gene (Maff2-related) from Tn*5252*-related ICEs	Replisome organizer	[30]
*tet*(O) fragment	*Streptococcus pyogenes* (fir.)	Ser	Internal site of a gene (SNF2 helicase) from Tn*5252*-related ICEs	RepA, ParB	[31]
Tn*6104*	*Clostridioides difficile* (fir.)	Ser	Internal site of *traG* (VirD4 CP) from Tn*5252* related ICEs	Replisome organizer, DnaC*,* TA	[32]
IME_*Sco1050_traG_site1*^f^	*Streptococcus constellatus* C1050 (fir.)	Ser	Internal site S1 of *traG* (VirD4 CP) from Tn*5252*-related ICEs	Replisome organizer	[26]
IME_*Sco1050_traG_site2*^f^	*Streptococcus constellatus* C1050 (fir.)	Ser	Internal site S2 of *traG* (VirD4 CP) from Tn*5252-*related ICEs	Replisome organizer	[26]
Tn*4451* ^f^	*Clostridium perfringens* (fir.)	Ser	Numerous sites (GA)		[33]
ATE-1	*Trueperella pyogenes* (act.)	Tyr	3′ end of *guaA* (GMP synthase)	TA	[34]
IncP island ^g^	*Brucella suis* (α)	Tyr	3′ end of *guaA* (GMP synthase)	RepA, antitoxin	[35]
Gisul2	*Pseudomonas aeruginosa* (γ) ^h^	Tyr	3′ end of *guaA* (GMP synthase)	RepA, RepC	[36]
IME_*SagNEM316_rplL*^f^	*Streptococcus agalactiae* (fir.)	Tyr	3′ end of *rplL* (L7/L12 ribosomal protein)		[26]
IME_*Sga2069_rpmE*^f^	*Streptococcus gallolyticus* (fir.)	Tyr	3′ end of *rpmE* (L31 ribosomal protein)	Rep_Trans	[26]
IME_*SSalJIM777_rpmG* ^f^	*Streptococcus salivarius* (fir.)	Tyr	3′ end of *rpmG* (L33 ribosomal protein)		[26]
IME*Sag-rpsI* ^g^	*Streptococcus agalactiae* HRC (fir.)	Tyr	3′ end of *rpsI* (S9 ribosomal protein)	Rep_Trans, TA	[37]
SGI1 ^g^	*Salmonella enterica* DT104 (γ)	Tyr	3′ end of *trmE* (tRNA modification GTPase)	Rep_3, TA	[38]
MGI*Vch*Hai6	*Vibrio cholerae* (γ)	Tyr	3′ end of *trmE* (tRNA modification GTPase)		[39]
MGI*Vch*Moz6	*Vibrio cholerae* (γ)	Tyr	3′ end of *yicC* (unknown)	RM II	[40]
MGI*Vch*USA1 ^f^	*Vibrio cholerae* (γ)	Tyr	3′ end of *yicC* (unknown)	2 TAs	[27]
MGI*Vfl*Ind1 ^f^	*Vibrio fluvialis* (γ)	Tyr	3′ end of *yicC* (unknown)		[27]
MGI*Vmi1*	*Vibrio mimicus* (γ)	Tyr	3′ end of *yicC* (unknown)		[41]
BcenGI2 ^f^	*Burkholderia cenocepacia* (β)	Tyr	3′ end of tRNAala gene	Rep_3, TA	[42]
IME_*SsuTL13-tRNAasn*	*Streptococcus suis* (fir.)	Tyr	3′ end of tRNAarg gene		[26]
IME_*SanC1051_tRNAarg*	*Streptococcus anginosus* (fir.)	Tyr	3′ end of tRNAasn gene		[26]
NBU1	*Bacteroides uniformis* (bac.)	Tyr	3′ end of tRNAleu gene	TA	[43]
MTn*Pi4*	*Prevotella intermedia* (bac.)	Tyr	3′ end of tRNAleu gene		[44]
IME_*Sag2603_tRNAlys* ^f^	*Streptococcus agalactiae* (fir.)	Tyr	3′ end of tRNAlys gene		[45]
Tn*6031*	*Sphingobacterium* sp. (bac.)	Tyr	3′ end of tRNApro gene		[46]
NBU2	*Bacteroides fragilis* (bac.)	Tyr	3′ end of tRNAser gene		[47]
IME_*SdyRE378_ebfC*^f^	*Streptococcus dysgalactiae* (fir.)	Tyr	5′ end of *ebfC* (nucleoid associated protein)		[26]
IME_*SanC238_tatD*	*Streptococcus anginosus* (fir.)	Tyr	5′ end of *tatD* (DNAse)		[26]
IME_*oriT* ^g^	*Streptococcus agalactiae* (fir.)	Tyr	*oriT* from Tn*916* and ICE*St3*-related ICEs		[48]
Tn*4555*	*Bacteroides vulgatus* (bac.)	Tyr	Two preferred sites		[49]
Tn*5520*	*Bacteroides fragilis* (bac.)	Tyr	AT-rich regions	None	[50]
Tn*6218*^g^	*Clostridioides difficile* (fir.)	Tyr	AT-rich regions		[51]
cLV25	*Bacteroides fragilis* (bac.)	Tyr	ND		[52]
MTn*Pi1*	*Prevotella intermedia* (bac.)	Tyr duo	TTAC NNNNN AA		[44]
MTn*Pi2*	*Prevotella intermedia* (bac.)	Tyr duo	TTGC NNNNN AA		[44]
MTn*Pi3*	*Prevotella intermedia* (bac.)	Tyr duo	TTAC NNNNN A/G A/G		[44]
Tn*4399*	*Bacteroides fragilis* (bac.)	ND	Numerous sites		[53]

^a^ This table includes all types of IMEs or putative IMEs that were found to excise and some elements that were not tested for excision (generally one per type). The activity of excision of underlined elements has not been demonstrated. The elements are sorted according to their integration type and their specificity of integration. ^b^ α, alphaproteobacteria; β, betaproteobacteria; γ, gammaproteobacteria; act., actinobacteria; bac., bacteroidetes; fir., firmicutes. ^c^ Int, integrase type. DDE, DDE transposase; Ser, serine recombinase; Tyr, tyrosine recombinase; Tyr duo, two distantly related tyrosine recombinases encoded by two tandem genes. ^d^ Integration sites are given only when numerous events of integration were analysed and/or when features characteristic of a site-specific integration were found. The specific integration in the target gene leads either to a disrupted target gene (internal site) or to a gene encoding an unchanged or almost unchanged tRNA or protein (3′ end, 5′ end). ND, not determined. **^e^** The element encodes proteins that could be involved in the maintenance of the excised element. Rep_Trans: protein involved in rolling circle replication (RCR) initiation. RepA, RepC, Rep_3, Replisome organizer: proteins involved in theta replication initiation. RM II: Type II restriction-modification. TA: toxin-antitoxin. ^f^ Published putative IME(s) that have closely related mobilization modules but have at least some different passenger gene(s) is(are) not mentioned in this table. ^g^ This name refers to a family of elements, some of which differing in their gene content. ^h^ Identical or very closely related IMEs are found in various species.

**Table 2:** Mobilization of IMEs and putative IMEs.

IME ^a^	Species (Division) ^b^	Mobilization Proteins Encoded by the IME ^c^		Mobilizing Element (CP, MPF) ^d^	Reference
		Relaxase	Others		
MTn*Sag1*	*Streptococcus agalactiae* (fir.)	None	None	Tn*916* (TcpA, FA)	[28]
tIS*Cpe8*	*Clostridium perfringens* (fir.)	None	None	Tn*916* (TcpA, FA)	[29]
GIE492 ^e^	*Klebsiella pneumoniae* (γ)	None		Proposed: ICE*Kp1* (VirD4, T)	[87]
MGI*Vfl*Ind1 ^e^	*Vibrio fluvialis* (γ)	None		ICE*Vfl*Ind1 and SXT (VirD4, F)	[27]
MGI*Vch*Hai6 ^e^	*Vibrio cholerae* (γ)	None	1 RAF	IncA/C plasmids (VirD4, F)	[39]
MGI*Vmi1* ^e^	*Vibrio mimicus* (γ)	None	1 RAF	IncA/C plasmids (VirD4, F)	[41]
SGI1 ^e,f^	*Salmonella enterica* (γ)	None	TraG, TraH, TraN	IncA/C plasmids (VirD4, F)	[38]
Gisul2 ^g^	*Pseudomonas aeruginosa* (γ)	None	TrbJ, TrbK, TrbL	Proposed: IncP plasmids (VirD4, T)	[36]
IME_*SsalCCHSS3_ND*	*Streptococcus salivarius* (fir.)	MobC	VirD4		[26]
NBU1	*Bacteroides uniformis* (bac.)	MobP		CTnERL and CTnDOT (VirD4, B); IncP plasmids (VirD4, T)	[43]
NBU2	*Bacteroides fragilis* (bac.)	MobP		CTnERL (VirD4, B); IncP plasmids (VirD4, T)	[47]
Tn*4555*	*Bacteroides vulgatus* (bac.)	MobP		CTn*341* (VirD4, B); IncP plasmids (VirD4, T)	[49]
cLV25	*Bacteroides fragilis* (bac.)	MobP	1 RAF	IncP plasmids (VirD4, T)	[52]
Tn*4399*	*Bacteroides fragilis* (bac.)	MobP	1 RAF	CTnDOT (VirD4, B); IncP plasmids (VirD4, T)	[88]
IncP island ^f^	*Burkholderia glumae* (β)	MobP	2 RAFs, TrbJ, TrbK, TrbL	Proposed: IncP plasmids (VirD4, T)	[89]
IME_*ScoC232_maff2_site 1*	*Streptococcus constellatus* (fir.)	MobP			[26]
IME*Sp2907* ^e^	*Streptococcus pyogenes* (fir.)	MobQ		Proposed: Tn*5252* superfamily (VirD4, FATA)	[30]
ATE-1	*Trueperella pyogenes* (act.)	MobV		IncP plasmids (VirD4, T)	[34]
Tn*5520*	*Bacteroides fragilis* (bac.)	MobV	None	IncP plasmids (VirD4, T)	[50]
Tn*6215*	*Clostridioides difficile* (fir.)	MobV		ND	[90]
Tn*4451* ^e^	*Clostridium perfringens* (fir.)	MobV		IncP plasmids (VirD4, T)	[91]
IME*Sag-rpsI* ^f,g^	*Streptococcus agalactiae* HRC (fir.)	MobV		pAMβ1 plasmid (VirD4, FATA)	[37]
*tet*(O) fragment	*Streptococcus pyogenes* (fir.)	MobV		Proposed: Tn*5252* superfamily (VirD4, FATA)	[31]
IME-*oriT* ^e,f,h^	*Streptococcus agalactiae* (fir.)	MobT		Proposed: Tn*916* and ICE*St3* (TcpA, FA)	[63]
IME_*Sag2603_tRNAlys*^e^	*Streptococcus agalactiae* (fir.)	MobT		Proposed: helpers with TcpA and FA	[26]
IME_*SsuBM407_tRNAleu*^e^	*Streptococcus suis* (fir.)	MobT	TcpA	Proposed: helpers with TcpA and FA	[26]
IME_*SdyRE378_ebfC*^e^	*Streptococcus dysgalactiae* (fir.)	PF01719		Proposed: helpers with TcpA and FA	[26]
IME_*SsalJIM8777_rpmG*^e^	*Streptococcus salivarius* (fir.)	PF01719	TcpA	Proposed: helpers with TcpA and FA	[26]
IME_*SsuTL13_rpsI*^e^	*Streptococcus suis* (fir.)	PF01719-helicase		Proposed: helpers with TcpA and FA	[26]
IME*_Seq35246_rpsI*^e^	*Streptococcus equi* (fir.)	PF01719-helicase	TcpA	Proposed: helpers with TcpA and FA	[26]
IME_*SanC238_tatD*	*Streptococcus anginosus* (fir.)	PHA00330		Proposed: helpers with TcpA and FA	[26]
IME_*SpnA45_tRNAleu*^e^	*Streptococcus pneumoniae* (fir.)	PHA00330	TcpA	Proposed: helpers with TcpA and FA	[26]
IME_*SiniSF1_ebfC*	*Streptococcus iniae* (fir.)	PF02407	TcpA	Proposed: helpers with TcpA and FA	[26]

^a^ This table includes all types of IMEs that were found to be mobilized *in trans* (generally one per type) and some putative ones. The activity of transfer of underlined elements has not been demonstrated. ^b^ α, alphaproteobacteria; β, betaproteobacteria; γ, gammaproteobacteria; act., actinobacteria; bac., bacteroidetes; fir., firmicutes. ^c^ The elements are sorted according to the relaxase family. MobC, MobP, MobQ, MobV: canonical relaxases. MobT, Rep_2, Rep_2—helicase, Viral-Rep, PHA00330: putative non-canonical relaxases related to RCR initiators. RAF: putative relaxosome accessory factor. TcpA: non-canonical coupling protein. TraG, TraH, TraN, TrbJ, TrbK, TrbL: MPF proteins. VirD4: canonical coupling protein. ^d^ The helper elements from heterologous bacteria are underlined. The coupling protein class and the MPF class of the helper element are given in brackets. ND: the IME was found to transfer in its native host, but the helper element has not been identified. Proposed: prediction of helpers could be made according to the IME characteristics. ^e^ Published putative IME(s) that have closely related mobilization modules but have at least some different passenger gene(s) is(are) not mentioned in this table. ^f^ This name refers to a family of elements, some of which differing in their gene content. ^g^ Identical or very closely related IMEs are found in various species. ^h^ The mobilization of the IME has not been tested but a recombinant plasmid carrying its mobilization module has been shown to be mobilized *in trans* in its native host or in a closely related bacterium.

**Table 3:** Cargo genes carried by IMEs and putative IMEs.

IME ^a^	Species (Division) ^b^	Size	Putative Cargo Genes		Reference
			Resistance Genes	Others	
ATE-1	*Trueperella pyogenes* (act.)	10.8	*tet*(W) (tetracycline)	TA, 3 unknown	[105]
Tn*6031*	*Sphingobacterium* sp. (bac.)	13.0	*tet*(X) (tetracycline), *aadS* (streptomycin)	5 unknown	[46]
*tet*(O) fragment	*Streptococcus pyogenes* (fir.)	13.4	*tet*(O) (tetracycline)	RNA polymerase sigma factor sigma-70, 5 unknown	[31]
IME*Sp2907*	*Streptococcus pyogenes* (fir.) *Streptococcus agalactiae* (fir.)	12.6	*erm*(TR) (macrolide, lincosamide, streptogramin)	11 unknown	[31] [101]
*ermF* region	*Bacteroides thetaiotaomicron* (bac.)	13.0	*ermF* (clindamycin, erythromycin)	4 unknown	[106]
tIS*Cpe8*	*Clostridium perfringens* (fir.)	2.0	*lnu*(P) (lincomycin)		[29]
MTn*Sag1* (tIS*Sag10*)	*Streptococcus agalactiae* (fir.)	1.7	*lnu*(C) (lincomycin)		[28]
IME_*GB00957_oriT*	*Streptococcus agalactiae* (fir.)	5.2	*lsa*(C) (lincosamide, streptogramin A and pleuromutilin)	2 unknown	[48]
NBU2	*Bacteroides fragilis* (bac.)	11.1	*linA_N2_* (lincomycin, clindamycin), *mefE_N2_* (erythromycin)	2 unknown	[47]
SGI1	*Salmonella enterica* (γ)	42.4	*aadA2* (streptomycin, spectinomycin), *floR* (chloramphenicol, florfenicol), *tet*(G) (tetracycline), *bla*_PSE-1_ (ampicillin), *sul1* (sulfonamides)	TA, 18 unknown	[38]
SGI1-V	*Proteus mirabilis* (γ)	42.9	*aacA4* (kanamycin, tobramycin, netilmicin, amikacin), *aadB* (kanamycin, gentamicin, tobramycin); *dhfrA1* (trimethoprim), *bla*_VEB-6_ (extended-spectrum cephalosporin), *sul1* (sulfonamides), *qnrA1* (quinolones)	20 unknown	[107]
SGI1-L	*Morganella morganii* (γ)	50.3	*tet*(G) (tetracycline), *floR* (chloramphenicol, florfenicol), *dhfrA_15_* (trimethoprim), *bla*_PSE-1_ (amoxicillin, clavulanate), *sul1* (sulfonamides)	11 unknown	[108]
MGI*Vch*Hai6	*Vibrio cholerae* (γ)	47.4	*aadA2* (streptomycin, spectinomycin), *floR* (chloramphenicol, florfenicol), *tet*(G) (tetracycline), *bla*_PSE-1_ (ampicillin), *sul1* (sulfonamides), *merEDAFPT* (mercury)	RM I, 6 unknown	[39]
IncP island	*Brucella suis* (α)	12.7		antitoxin, 2 unknown	[35]
IncP island ^c^	*Burkholderia glumae* (β), *Acidovorax avenae* (β)	14.1	*aac(2*′*)-IIa* (kasugamycin)	antitoxin, 3 unknown	[89]
Tn*4453* ^c^	*Clostridioides difficile*	6.3	*catD* (chloramphenicol)	2 unknown	[69]
Tn*4555*	*Bacteroides vulgatus*	12.2	*cfxA* (cefoxitin)	2 unknown	[67]
GI*sul2*	*Pseudomonas aeruginosa* (γ)	15.4	*sul2* (sulphonamide), *arsBCHR* (arsenate/arsenite)	TA, 4 unknown	[36]
IME_*Sag2603_tRNAlys*	*Clostridioides difficile* (fir.)	10.5		ABC transporter of the drug resistance transporter subfamily, 8 unknown	[48,84]
IME_*SagA909_tRNAlys*	*Clostridium perfringens* (fir.)	8.3		Intracellular protease, 7 unknown	[48,84]
IME*Sag-rpsI*	*Streptococcus agalactiae* (fir.)	9.1		TA, 6 unknown	[37]
IME_*Sag2603_rpsI*	*Streptococcus agalactiae* (fir.)	9.0	*arsR* (arsenate reductase)	TA, 5 unknown	[84]
IME*_18RS21_oriT*	*Streptococcus agalactiae* (fir.)	6.4	*mer*A, *mer*R (mercury)	3 unknown	[48]
MTn*Pi2*	*Prevotella intermedia* (bac.)	16.6		LuxR family transcriptional regulator, 9 unknown	[44]
MTn*Pi3*	*Prevotella intermedia* (bac.)	18.4		2 ABC transporter components, 6 unknown	[44]
MTn*Pi4*	*Prevotella intermedia* (bac.)	12.4		Subtilase-like protease, 7 unknown	[44]
MGI*Vch*USA1 ^c^	*Vibrio cholerae* (γ)	22.0		two TA, 10 unknown	[27]
MGI*Vch*Moz6	*Vibrio cholerae* (γ)	19.7		RM II, 7 unknown	[40]
MGI*Vmi1*	*Vibrio mimicus* (γ)	16.5		RM III, 10 unknown	[87]
GIE492	*Klebsiella pneumoniae* (γ)	22.3		MccE492 microcin (bacteriocin), 7 unknown	[32]
Tn*6104*	*Clostridioides difficile* (fir.)	15.6		Lantibiotic synthesis (bacteriocin), T/A, 7 unknown	[61]
Tn*5520*	*Bacteroides fragilis* (bac.)	4.7			[87]

TA, toxin-antitoxin systems; RM, restriction modification systems genes type I, II or III; unknown, genes with unknown or unannotated functions that could correspond either to cargo genes or to genes involved in IME transfer or maintenance. The function of underlined genes has not been demonstrated. ^a^ This table includes some IMEs and putative ones whose gene content is known. ^b^ α, alphaproteobacteria; β, betaproteobacteria; γ, gammaproteobacteria; act., actinobacteria; bac., bacteroidetes; fir., firmicutes. ^c^ Identical or very closely related IMEs are found in various species.
